# Obsessive-Compulsive Disorder in children. A case report

**DOI:** 10.1192/j.eurpsy.2022.1112

**Published:** 2022-09-01

**Authors:** R. Galerón, M. Huete Naval, E. Exposito Duran, F. Mayor Sanabria, C. Regueiro Martín-Albo, F. García-Escribano Martín

**Affiliations:** 1 Hospital Clínico San Carlos, Institute Of Psychiatry And Mental Health, Madrid, Spain; 2 Hospital Clínico San Carlos, Instituto De Psiquiatría Y Salud Mental, Madrid, Spain

**Keywords:** Children, Specific phobia, Autism Spectrum Disorder, obsessive-compulsive disorder

## Abstract

**Introduction:**

We present a 9-year-old girl with celiac disease who attends a Health Center referred by her pediatrician for rituals. Her mother describes rituals from early childhood that have been intensified by the death of her grandmother from pancreatic cancer. Since then, thoughts of gluten contamination and behaviors aimed at avoiding such contamination have increased. For example, not using the common household towel and not eating until all the guests have washed their hands. If the patient does not carry out these actions, she presents significant discomfort, crying and screaming until it is done. In addition, such behaviors take up a significant amount of time.

**Objectives:**

To review the literature of Obsessive-Compulsive Disorder (OCD) in children.

**Methods:**

Literature review of scientific articles searching in Pubmed. We considered articles in English and Spanish.

**Results:**

Cognitive Behavioral Therapy and Sertraline up to 50mg were started. After several weeks the frequency of behaviors aimed at avoiding gluten contamination begins to decrease; as well as the anguish if these are not carried out.

**Conclusions:**

OCD in childhood can present characteristics that differentiate it from OCD in adulthood, such as difficulty detecting obsessions and that children do not usually consider thoughts as unreal or excessive. Therefore, it is a real challenge, having to carry out an adequate differential diagnosis with other entities such as specific phobias, for adequate subsequent management.

**Disclosure:**

No significant relationships.

